# Proteomic Modeling for HIV-1 Infected Microglia-Astrocyte Crosstalk

**DOI:** 10.1371/journal.pone.0002507

**Published:** 2008-06-25

**Authors:** Tong Wang, Nan Gong, Jianuo Liu, Irena Kadiu, Stephanie D. Kraft-Terry, R. Lee Mosley, David J. Volsky, Pawel Ciborowski, Howard E. Gendelman

**Affiliations:** 1 Center for Neurovirology and Neurodegenerative Disorders, Department of Pharmacology and Experimental Neuroscience, Omaha, Nebraska, United States of America; 2 Molecular Virology Division, St. Luke's-Roosevelt Hospital Center and Columbia University Medical Center, New York, New York, United States of America; 3 Institute for Tissue Transplantation and Immunology, Jinan University, Guangzhou, Guangdong, China; AIDS Research Center, Chinese Academy of Medical Sciences and Peking Union Medical College, China

## Abstract

**Background:**

HIV-1-infected and immune competent brain mononuclear phagocytes (MP; macrophages and microglia) secrete cellular and viral toxins that affect neuronal damage during advanced disease. In contrast, astrocytes can affect disease by modulating the nervous system's microenvironment. Interestingly, little is known how astrocytes communicate with MP to influence disease.

**Methods and Findings:**

MP-astrocyte crosstalk was investigated by a proteomic platform analysis using vesicular stomatitis virus pseudotyped HIV infected murine microglia. The microglial-astrocyte dialogue was significant and affected microglial cytoskeleton by modulation of cell death and migratory pathways. These were mediated, in part, through F-actin polymerization and filament formation. Astrocyte secretions attenuated HIV-1 infected microglia neurotoxicity and viral growth linked to the regulation of reactive oxygen species.

**Conclusions:**

These observations provide unique insights into glial crosstalk during disease by supporting astrocyte-mediated regulation of microglial function and its influence on the onset and progression of neuroAIDS. The results open new insights into previously undisclosed pathogenic mechanisms and open the potential for biomarker discovery and therapeutics that may influence the course of HIV-1-mediated neurodegeneration.

## Introduction

Astrocytes comprise more than 50% of total cells in brain and serve pivotal homeostatic and regulatory functions for maintaining blood-brain barrier and neuron integrity [1], [2]. This is made possible by their functional roles in regulating extracellular glutamate, supporting a glial-neuronal network, controlling neuronal physiologic activities, promoting neurogenesis and secretion of neurotrophins [3]–[5]. Growing evidence suggests that human immunodeficiency virus type one (HIV-1) infection of the central nervous system (CNS) may affect some of these functions and contribute to neuropathogenesis in distinct pathways (reviewed in [6]–[8]).

Astrocytes serve as natural host cells for HIV-1 particularly in advanced brain disease [9]–[13]. Moreover, HIV and gp120 bind efficiently to astrocytes [14]–[18], but during both in vitro and in vivo HIV infection only in a small proportion of infected astrocytes can be detected [11], [13], [14], [16], [19]; a restriction that has recently been attributed to absence of CD4 on astrocytes and limited virus entry [20]–[22]. Productive infection of human astrocytes with HIV-1 has significant effects on cell physiology in vitro [23], [24] and is associated with measurable neuropathology in a mouse model [25]. Thus infected astrocytes, although infrequent, can have localized pathogenic effects. At another level, as part of brain parenchyma, astrocytes are likely exposed continuously to HIV-1 particles, viral gp120, Tat proteins, cytokines, and other substances secreted by HIV-1-infected macrophages and microglia. Studies in vitro indicate that many of these products significantly modulate astrocyte physiology, which in turn can alter essential interactions of astrocytes with other cells in the brain, particularly neurons. For example, exposure of cultured astrocytes to HIV and gp120 induces extensive changes in cellular gene expression [8], [26]–[28] and impairs transport of extracellular glutamate by astrocytes [29], [30]; a defect which may lead to neuronal death by glutamate excitotoxicity [31]. HIV-1, recombinant gp120, and viral transactivator Tat activate astrocytes to secrete the pro-inflammatory cytokines TNF-α, IL-6, and IL-1β; the pro-inflammatory chemokines MCP-1 and IP-10; and neurotoxic nitric oxide (NO) [11], [30]–[38], all of which could contribute to the overall inflammatory environment in the brain. Glutamate uptake can also be impaired by intracellular expression of recombinant Tat or exposure of astrocytes to TNF-α [32], [33]. Additional insight into physiological effects of Tat on astrocytes was obtained in recent studies using proteomics, which revealed decreased synthesis of products such as phosphatase 2A inhibitor, the mitochondrial enzyme isocitrate dehydrogenase, and α-tubulin/vimentin with concomitantly increased levels of heme oxygenase 1, heat shock protein 70, and iNOS [34], [35]. Overall, these findings suggest that astrocytes rendered dysregulated by exposure to HIV-1 in the brain have the capacity to injure or impair neurons. Because both HIV-1 binding and native infection can affect astrocyte function in vitro and in vivo [24], [25], [30], astrocytes possess a pathogenic potential that exceeds their susceptibility to HIV-1 infection.

In contrast to the large body of work on astrocyte-neuronal interactions, there is surprisingly little information on the potential cross-talk between astrocytes and macrophages/microglia in the context of HIV infection. Yet until recently, HIV mediated neuropathogenesis was considered to revolve solely around metabolic processes induced by viral infection and activated mononuclear phagocytes (MP: perivascular macrophages and microglia) [36]–[38]. The role of brain MP in the pathobiology of neuroAIDS rests as a cell source for pro-inflammatory neurotoxic products and as a continuous reservoir for productive viral replication [39]–[41]. Activated astrocytes can exert both protective and detrimental effects on neurons [42], [43]. Astrocytes have been shown to accelerate neurotoxic brain MP activities and regulate such responses [19], [25], [44], [45], but how and under what conditions this occurs and the intercellular effects remain unknown. Although it is well known that HIV-1 replication and innate immune responses in the brain are limited in the early stages of viral infection [40], [41], [46], the relative contributions of cytotoxic T lymphocytes or glial cells to this process are unclear.

The present study posits that astrocyte–microglial crosstalk contributes to control of HIV mediated neuropathogenesis. Previous research performed in our laboratories demonstrated that macrophage neurotoxicity was affected in the setting of HIV-1 infection and astrocyte co-cultivation [47]. Astrocytes may exert regulatory roles in disease depending on the MP activation state [42], [43]. These studies focused on the cellular control mechanisms that influence cognitive and motor dysfunctions in HIV-1-infected individuals, but left unresolved a wide range of questions that could only now be addressed through the advent of proteomics, live cell confocal microscopy, and new murine model systems for study of HIV infection. For the first time, we were able to conduct our experiments with HIV-infected isogeneic primary mouse macrophages and astrocytes, thus minimizing the heterogeneity of cellular responses observed when using human cells from multiple donors. This approach build on recent findings demonstrating that primary mouse cells including macrophages, lymphocytes, and astrocytes can support efficient HIV replication when infected with HIV carrying envelope proteins that recognize mouse cell receptors, such as HIV pseudotyped with vesicular stomatitis virus (VSV)-G protein [48], [49] or recombinant HIV expressing MuLV gp80 envelope, EcoHIV [50]–[52]. Using VSV/HIV for infection of mouse cells and proteomic approaches to investigate the effects of astrocytes on HIV-1-infected microglia, we show herein that microglial F-actin polymerization, filament formation, and cell migration are altered by astrocyte secretions. Astrocytes modulate microglial activation by regulating reactive oxygen species and cell death pathways that regulate viral growth. Such astrocyte-microglial crosstalk profoundly affects microglial structural and secretory functions that in turn affect the pathobiology of disease leading to disease onset and neuroAIDS progression in an infected human host.

## Results

### Neurotoxic activities of HIV-1/VSV pseudotype infected microglial

**Figure 1:**
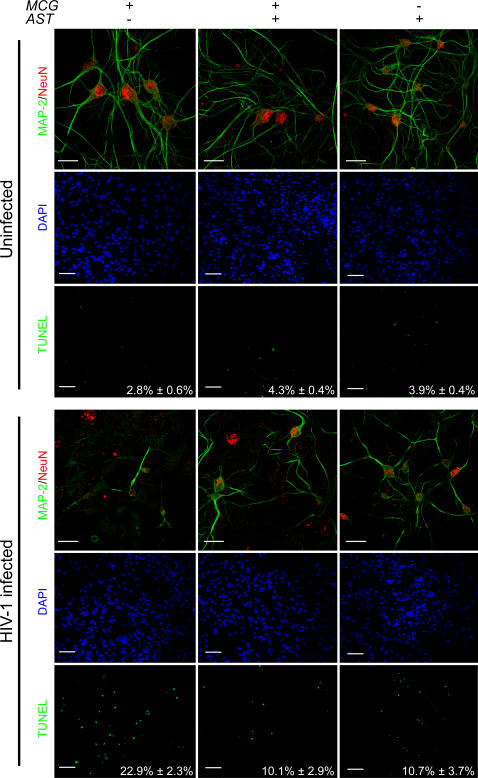
Neurotoxic activities of culture fluids from HIV-1/VSV infected microglia attenuated by microglia-astrocyte co-cultivation. Mouse neurons were cultured for 10 d and exposed to the 20% conditioned media (CM) from uninfected or HIV-1/VSV infected microglia and/or astrocytes for 24 h. MAP-2 (green) and NeuN (red) expression by neurons was visualized by immunohistochemical staining. Characteristic neurotoxicity, including released nuclear materials and broken neurites, were observed in neurons treated with CM from HIV-1/VSV infected microglia alone. Percentages of apoptotic neurons were evaluated by the ratio of TUNEL^+^ (green) neurons to DAPI^+^ (blue) cells. The results are depicted as a mean percentage of apoptotic cells±SEM of three experiments. Significant reduction of percentage of apoptotic cells were observed in HIV-1/VSV infected co-cultures and astrocyte groups, compared with those of neurons cultured in microglial CM alone (n = 3 determines/group, p<0.01, determined by one-way ANOVA analysis and Tukey's multiple comparison post-hoc tests). Bars for MAP-2/NeuN, 20 µm; bars for DAPI and TUNEL, 50 µm.

To preclude inter-strain variation, microglia and astrocytes were obtained from identical inbred mouse strains. HIV-1 infection was facilitated in murine cells by circumventing necessary HIV-1 co-receptors by using a HIV-1/VSV pseudotyped virus. This model provided productive infection of microglia and microglia-astrocyte cross-talk responses in the absence of genetic mismatches. To substantiate the capacities of microglial and astrocyte secretory responses to affect neuronal toxicity, we measured neuronal cell death in primary neuron cultures after 24 hour exposure to conditioned media (CM) from cultures of infected microglia alone, astrocytes alone, or infected microglia co-cultured with astrocytes at a ratio of 1∶2 in transwell systems (Fig. 1). Expression of microtubule associate protein-2 (MAP-2, green) and the neuronal nuclei-specific protein, neuron-specific nuclear protein (NeuN, red) demonstrated that >98% of the cells were indeed neurons. After culture in the presence of CM from uninfected microglia and/or astroctyes, the levels of apoptotic neurons were relatively low (2.8%–4.3%) as determined by the percentage of neurons stained by terminal uridine deoxynucleotidyl transferase dUTP nick end labeling (TUNEL, green) and the total number of neuronal nuclei stained with DAPI. In contrast, CM from infected microglia increased neurotoxicity as shown by the 10-fold increase (22.9%) compared to CM from uninfected microglia. However, this neurotoxic response was attenuated with CM from infected microglia co-cultured with astrocytes as demonstrated by a significant diminution (>2 fold, 10.1%) of apoptotic neurons (n = 5, p<0.01).

### HIV-1/VSV infected microglial proteome

**Figure 2:**
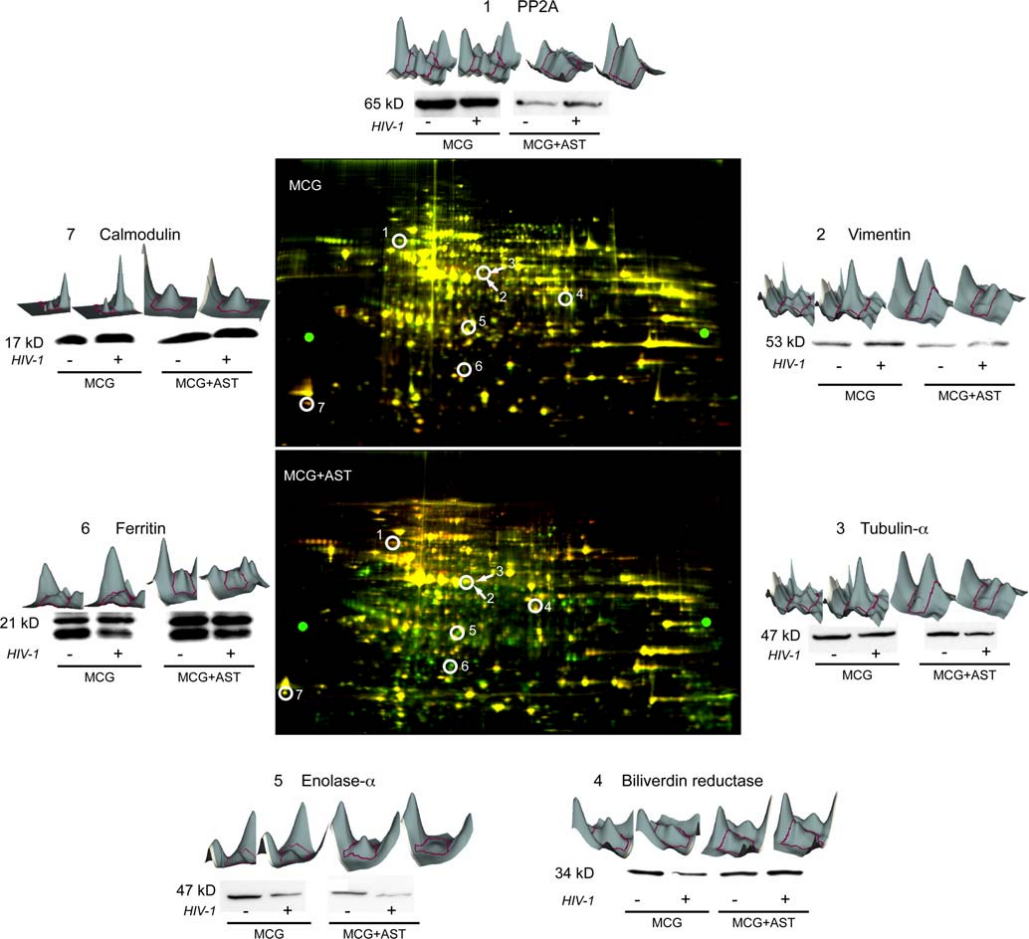
Proteome of HIV-1/VSV infected microglia (MCG) and MCG co-cultured with astrocytes (AST). DiGE analyses of lysates of uninfected and HIV-1/VSV infected microglia cultured alone (MCG, upper gel) or in the presence of astrocytes (MCG+AST, lower gel) Proteins from uninfected microglia lysates are labeled with Cy3 (green), whereas those from HIV-1/VSV infected microglia are labeled with Cy5 (red). After merging, proteins exhibiting no changes appear yellow in the DiGE image, down-regulated proteins in green, and up-regulated proteins in red. Three-dimensional DeCyder interpretation for seven representative proteins identified by mass spectrometry and corresponding Western blot analysis are shown around the DiGE images. Each pair of protein spots (Cy3-and Cy5-labeled) are 3D views of their relative peak volumes. The peak area comprising the entire volume represents the distribution of the protein spot in the gel, whereas the volume correlates to the protein concentration. The consecutive numbers of spots located in the respective DiGE images correspond to the protein volumes shown.

We next evaluated the proteome of the virus-infected microglia to determine the protein compositions associated with the observed neurotoxic responses. We reasoned that the microglial proteome was affected by HIV-1 infection and that regulation of specific proteins was linked to microglial neurotoxic activities and HIV-1 induced neurodegeneration. To test this idea, we employed a 2D difference gel electrophoresis (DIGE) proteomics platform to assess changes of the microglial proteome and its affects by astrocyte co-cultivation (Fig. 2). Following cultivation of uninfected or virus-infected microglia in the presence or absence of astrocytes, lysates from uninfected and infected microglia were labeled with the N-hydroxy-succinimidyl ester of carboxycyanine (Cy3) and 1-(5-carboxypentyl)-1-methylindodi-carbocyanine halide (Cy5), respectively, and a mixture of lysates from both groups were labeled by 1-(5-caboxypentyl)-1-propylindocarbocyanine halide (Cy2).

From virus-infected microglial lysates cultured in the absence of astrocytes, 2116 spots were digitally detected, of which 77 spots were differentially expressed as determined by either a 50% increase or decrease in protein spot volume compared with those from uninfected microglia. Those spots were robotically selected and excised, digested with trypsin, and subjected to analysis by liquid chromatography-tandem mass spectrometry (LC/MS/MS) (Fig. 2). From these spots, 39 and 24 proteins were confirmed to be up- or down-regulated, respectively, in infected microglia, of which 14 were identified. These proteins were grouped into functional classes as structural, regulatory, or enzyme according to the description provided by the ExPASy Proteomics Server Classification (http://ca.expasy.org/) (Table 1). Proteins, including cofilin and MARCKS, proliferation marker PCNA, and the energy “linked” protein, pyruvate kinase, were down-regulated after viral infection, whereas calmodulin was up-regulated.

**Table 1:** Changes in the proteome of HIV-1/VSV infected microglia.

Regulation and Protein Class	Protein Name	Swissprot Access No.	Fold Change^a^	M.W. Da	pI^b^	Peptides^c^
Up-regulated						
Structural	LOC683313 protein	Q4FZU2	1.5	59249	8.03	3
	Myotrophin	P62774	1.5	12861	5.28	2
Regulatory	Tyki protein	Q3U5Q7	1.9	46835	6.09	6
	Proteasome activator complex subunit 1	P97371	1.6	28673	5.83	4
	Calmodulin	P62204	1.7	16838	4.09	7
Enzymes	Adenine phosphoribosyltransferase	P08030	1.5	19736	6.31	2
Down-regulated						
Structural	40S ribosomal protein SA	P14206	1.7	32838	4.74	2
	Cofilin-1	P18760	1.6	18560	8.22	3
	Beta-actin FE-3	Q99NC5	1.6	14983	5.65	2
	Proliferating cell nuclear antigen	P17918	1.7	28785	4.66	2
Regulatory	Gelsolin	P13020	1.6	85942	5.83	5
	MARCKS (myristylated alanine-rich protein kinase C substrate)	P26645	1.5	29661	4.34	7
	Heterogeneous nuclear ribonucleoprotein K	Q5FWJ5	1.5	51028	5.19	3
Enzymes	Pyruvate kinase isozyme M2	P52480	1.6	57845	7.42	2

a Compared with uninfected microglia.

b Theoretical isoelectric point calculated by Swissprot database at http://ca.expasy.org/sprot/.

c Number of peptides detected by mass spectrometry for each identified protein.

In contrast, lysates from infected microglia co-cultured with astrocyte showed 149 and 360 spots that were up- or down-regulated proteins, respectively from a total of 2043 proteins (Fig. 2). Three hundred and seventy-six spots were picked, digested and analyzed by LC/MS/MS; from which 68 proteins were identified and similarly classified (Table 2). After infection, serine/threonine phosphatase 2A (PP2A) and biliverdin reductase were up-regulated; whereas tubulin-α, enolase-α and ferritin were down-regulated (Fig 2). Expression levels of 7 proteins identified by mass spectrometry were validated by Western blot assays in 3 independent experiments, each with triplicate determinations. Interestingly, cross comparison of the two different cell culture models revealed PP2A levels in microglia co-cultured with astrocytes were reduced compared to those in single cell type cultures (n = 3, p<0.01) (Fig 2).

**Table 2:** Astrocyte effect on the proteome of HIV-1/VSV infected microglia.

Regulation and Protein Class	Protein Name	Swissprot Access No.	Fold Change^a^	M.W. Da	*p*I^b^	Peptides^c^
Up-regulated						
Structural	Dynein	O88487	1.6	68393	5.16	9
	Moesin	P26041	1.7	67738	6.00	7
	Lamin A	Q3U733	2.2	72427	6.54	5
	Lamin B2	P21619	1.6	67029	4.25	6
	Cytovillin	P26040	1.7	69434	6.00	4
	Microtubule plus end-directed kinesin motor 3A	P28741	1.9	80168	6.16	3
	L-plastin	Q61233	1.8	70149	5.20	3
	Radixin	P26043	1.5	68525	5.85	8
	GPI-anchored protein p137	Q60865	1.8	73547	5.24	4
Regulatory	HSP70 protein, mitochondrial	P38647	1.9	68525	5.91	9
	Heterogeneous nuclear ribonucleoprotein M	Q9D0E1	1.5	77648	9.01	3
	Coronin-7	Q9D2V7	1.6	100812	5.13	5
	Vacuolar ATP synthase catalytic subunit A	P50516	1.6	68267	5.13	14
	Sorting nexin-1	Q9WV80	1.7	58951	4.25	13
	MARCKS (myristylated alanine-rich protein kinase C substrate)	P26645	1.7	29644	4.34	6
	Protein kinase C and casein kinase substrate in neurons protein 2	Q9UNF0	1.8	55738	5.08	2
	Major vault protein	Q9EQK5	1.9	95951	5.43	9
	Programmed cell death 6-interacting protein	Q9WU78	2.1	96009	6.15	4
	DEAD (Asp-Glu-Ala-Asp) box polypeptide 1	Q922B8	1.5	82499	6.94	4
	Wiskott-Aldrich syndrome protein family member 2	Q8BH43	1.6	54073	5.38	4
	Rap2-interacting protein x	Q9D394	1.7	53006	5.36	3
	Gelsolin	P13020	1.7	85900	5.83	10
	Rho GTPase-activating protein 25	Q8BYW1	1.7	73382	6.03	7
Enzymes	Methylenetetrahydrofolate dehydrogenase	Q922D8	1.7	10099	6.68	9
	Ubiquitin carboxyl-terminal hydrolase 5	Q3U4W8	2.6	93354	4.94	10
	Transitional endoplasmic reticulum ATPase	Q01853	1.8	89307	5.14	7
	Serine/threonine-protein phosphatase 2A 65 kDa regulatory subunit A alpha isoform (PP2A)	Q76MZ3	1.8	65150	5.00	4
	Aconitase	P28271	3.0	82652	6.64	11
	Thimet oligopeptidase 1	Q8K2D4	1.7	78026	5.67	3
	Vacuolar ATP synthase catalytic subunit A	P50516	1.6	68267	5.13	14
	NADH dehydrogenase (ubiquinone) Fe-S protein 1	Q5SUH2	1.6	79748	5.51	5
	Biliverdin reductase A	Q9CY64	1.6	33524	6.53	6
Down-regulated						
Structural	Annexin A2	P07356	3.3	38676	7.55	3
	Annexin A1	P10107	3.0	38734	6.97	6
	Annexin A3	O35639	2.5	36370	5.33	3
	Alpha-tubulin 6	P68373	2.3	49909	4.96	7
	Tropomyosin-3	P21107	1.6	32862	4.68	5
	40S ribosomal protein S14	P62264	2.5	16272	10.10	2
	Beta-actin	P60710	2.9	41737	5.29	2
Regulatory	Coactosin-like protein	Q9CQI6	1.8	15944	5.28	3
	Glia maturation factor beta	Q9CQI3	1.9	16736	5.07	6
	Rho, GDP dissociation inhibitor (GDI) beta	Q5M860	1.9	22850	4.98	3
	Galectin-3	P16110	2.5	27366	8.47	
	14-3-3 zeta	P63101	3.0	27771	4.73	5
	Kinesin light chain 4	Q9DBS5	1.7	68612	5.76	2
	Regulating synaptic membrane exocytosis protein 2 [Fragment]	Q9JIS1	1.8	175803	9.45	9
	Ferritin light chain 1	P29391	2.4	20802	5.66	6
	5-hydroxytryptamine 2B receptor	Q02152	1.9	56508	9.05	7
	GRP1 binding protein [Fragment]	Q920B1	4.1	119873	8.82	
	Microtubule-associated protein RP/EB family member 1	Q61166	2.4	30015	5.12	2
	Adenomatous polyposis coli protein [Fragment]	Q61315	2.0	310898	7.44	5
	Alcohol dehydrogenase [NADP+]	Q9JII6	3.7	36432	6.90	7
	Fatty acid binding protein 4, adipocyte	P04117	1.9	14650	8.53	2
Enzymes	Nicotinamide nucleotide transhydrogenase	Q61941	2.4	113838	7.53	5
	Sarcoplasmic reticulum 2+-Ca-ATPase	P11507	3.1	114768	5.23	4
	Phenylalanyl-tRNA synthetase, mitochondrial	Q99M01	1.6	52303	6.68	5
	Triosephosphate isomerase	P48500	4.3	26848	6.89	10
	Calpain-12	Q9ER56	2.1	80588	5.91	9
	Glutathione S-transferase Mu 1	P10649	1.8	25822	8.33	3
	Fructose-bisphosphate aldolase A	P05064	3.3	39200	8.30	4
	Peroxiredoxin-1	P35700	3.0	22176	8.26	7
	Alpha-enolase	P17182	2.0	47140	6.37	6
	Cathepsin A	P16675	2.0	53843	5.56	2
	Lysozyme	P08905	1.6	16689	9.11	2
	Tyrosine 3-monooxygenase/tryptophan 5-monooxygenase activation protein, beta	A2A5N2	2.1	28086	4.77	12
	Heat-responsive protein 12	P52760	3.1	14255	8.73	2
	Phospholipase C, beta 4	Q6P8L2	2.0	53261	5.47	5
	Proteasome (prosome, macropain) subunit, alpha type 3	Q58EV4	3.0	28405	5.29	2

a Compared with uninfected microglia.

b Theoretical isoelectric point calculated by Swissprot database at http://ca.expasy.org/sprot/.

c Number of peptides detected by mass spectrometry for each identified protein.

### Microglial networks

**Figure 3:**
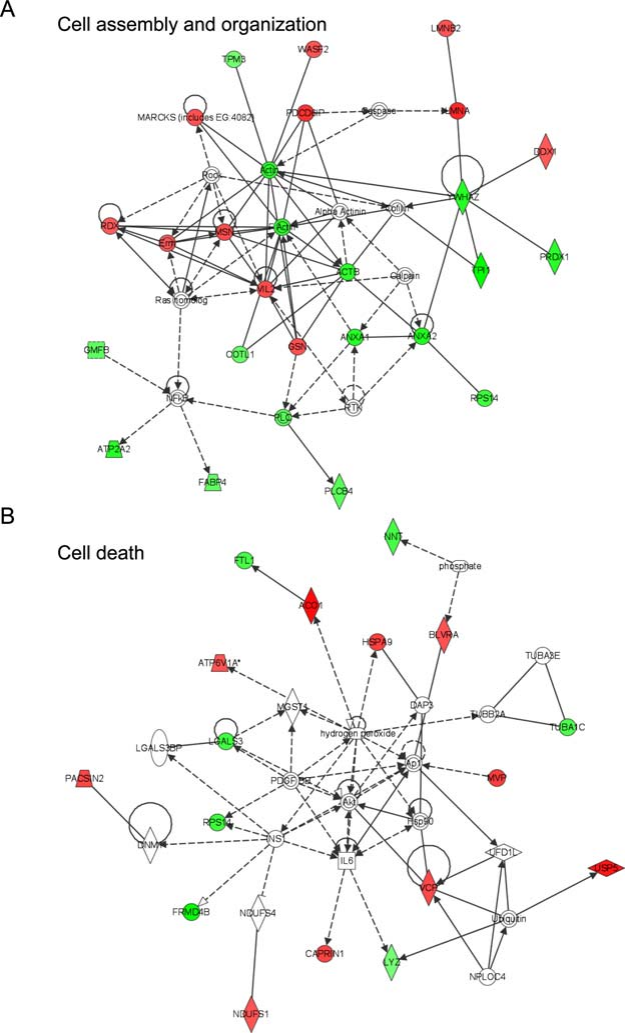
Ingenuity Pathway Analysis. Dynamic pathway/network modeling of the microglial proteome was afforded by integration of protein/gene data to the Ingenuity Pathway Analysis software of Ingenuity® Systems. Generated pathways show significant correlations with cellular assembly and organization (A) and cell death (B) networks. Interactions between proteins are shown by lines, whereas the lines with arrows represent direct interactions and no-arrow lines indicate binding only. Solid lines show the direct interaction, while the broken lines show indirect interaction. Nodes are represented by shapes and colors. Functions are indicated by shapes: diamonds for enzymes, squares for cytokines, rectangles for ligand dependant nuclear factors, triangles for kinases, ovals for transcription regulators, trapezoids for transporters, and circles indicate other interactions. Red color nodes represent the microglial proteins which are up-regulated upon HIV-1/VSV infection, while the green color nodes represent down-regulated proteins. Nodes without color represent proteins not input by user, but interpreted by the database as highly probable interactions within the network. The abbreviation used are: ACO1, aconitase; ACTB, beta-actin; ANXA1, annexin A1; ANXA2, annexin A2; APC, adenomatous polyposis coli protein; ATP2A2, sarcoplasmic reticulum 2+-Ca-ATPase; ATP6V1A, vacuolar ATP synthase catalytic subunit A; BLVRA, biliverdin reductase A; CAPRIN1, GPI-anchored protein p 137; COTL1, coactosin-like protein; DDX1, dead (Asp-Glu-Ala-Asp) box polypeptide 1; FABP4, fatty acid binding protein 4; FRMD4B, GRP1 binding protein; FTL1, ferritin light chain 1; GMFB, glia maturation factor beta; GSN, gelsolin; HSPA9, heat shock protein 70; LGALS3, galectin-3; LYZ, lysozyme; LMNA, lamin A; LMNB2, lamin B2; MARCKS (includes EG∶4082), myristylated alanine-rich protein kinase C substrate; MAPRE1, microtubule-associated protein; MSN, moesin; PDCD6IP, programmed cell death 6-interacting protein; MVP, major vault protein; NDUFS1, NADH dehydrogenase (ubiquinone) Fe-S protein 1; NNT, nicotinamide nucleotide transhydrogenase; PACSIN2, protein kinase C and casein kinase substrate in neurons protein 2; PLCB4, phospholipase C beta 4; PRDX1, peroxiredoxin-1; RDX, radixin; RPS14, 40S ribosomal protein S14; TPI1, triosephosphate isomerase; TPM3, tropomyosin-3; TUBA1C, alpha-tubulin 6; USP5, ubiquitin carboxyl-terminal hydrolase 5; VCP, transitional endoplasmic reticulum ATPase; VIL2, cytovillin; WASF2, Wiskott-Aldrich syndrome protein family member 2; YWHAZ, tyrosine 3-monooxygenase/tryptophan 5-monooxygenase activation protein beta.

We next examined the biological networks of microglial proteins affected by astrocytes and viral infection via dynamic pathway modeling (Ingenuity Pathway Analysis (IPA), Ingenuity® Systems, www.ingenuity.com). The 68 identified microglial proteins (Table 2) from the microglia-astrocyte co-culture group were searched with the corresponding Swissprot access numbers for their exact gene counterparts in IPA. The gene counterparts and their respective levels of changes were uploaded to the IPA module. IPA generated 2 high-scoring networks for pathways associated with cell assembly and organization, scoring 51 and involving 26 proteins (Fig. 3A) and cell death, scoring 34 and involving 17 proteins (Fig. 3B).

Interestingly, for regulated proteins associated with cell assembly and origination, 2 nodes of interest involving actins and NFκB were uncovered (Fig. 3A). Cell locomotion involves a cyclical process that requires cell protrusions at its leading edge and retractions at its trailing edge. Actin plays crucial roles in both processes. Microglial actins in astrocyte-co-cultures were down-regulated after HIV-1/VSV infection and the proteins related to actin binding, polymerization and stability, including MARCKS, moesin, Wiskott-Aldrich syndrome protein (WAS) and gelsolin, were all up-regulated. The down-regulation of these proteins suggests a less migratory phenotype after infection (Fig. 3A). Additionally, activation of NFκB is essential for microglia activation, however, direct agonists, glia maturation factor beta (GMFB) and phospholipase C beta 4, were down-regulated also suggesting a down-regulated phenotype associated with HIV-1/VSV infection.

IPA indicated that 8 of the microglial protein changes in infected microglia were related to increased cell death in eukaryotic cells (Fig 3B). Those down-regulated proteins included annexin A1, adenomatous polyposis coli protein, sarcoplasmic reticulum 2+-Ca-ATPase, enolase-α, GMFB and galectin-3; while up-regulated proteins included programmed cell death 6-interacting protein and PP2A. Interestingly, 6 of the protein changes associated with attenuation of cell death were down-regulated including galectin-3 and peroxiredoxin-1, whereas gelsolin, moesin, cytovillin and programmed cell death 6-interacting protein were up-regulated.

### Cytoskeletal transformation

We next performed morphological and immunochemical quantification to validate findings indicated by our pathway modeling of infected microglia-astrocyte (MCG-AST) interactions. Immunohistochemistry for expression of tubulin-α was used to assess microglial migratory morphologies that included protrusion formation and tailing structure, while assessment of F-actin expression was utilized to observe polarization; all essential features necessary for cell locomotion [53]. In addition, expression of vimentin and ionized calcium binding adaptor molecule 1 (Iba-1) was assessed to determine cell activation and purity. TNF-α was used as positive control for inducing activation and locomotion of microglia.

**Figure 4:**
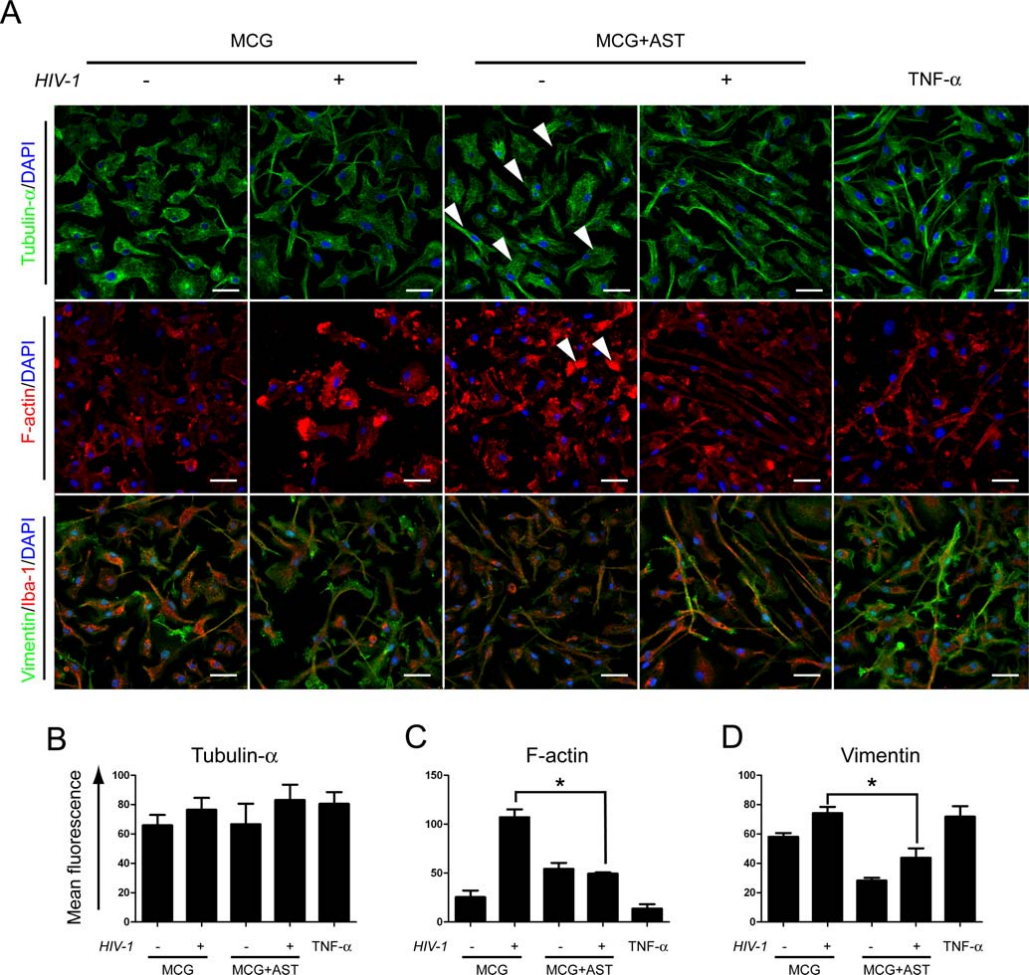
Immunohistochemical validation of proteomic profiling. Uninfected or HIV-1/VSV infected microglia were cultured for 24 h in the absence (MCG) or presence of astrocytes (MCG+AST). This arbitrary time point was chosen as it reflected dynamic changes in cell-cell interactions. Uninfected microglia cultured in the presence of 20 µg/ml TNF-α for 6 hours served as an activated microglia control. (A) Microglia were stained for the expression of the cytoskeletal proteins tubulin-α (green) and F-actin (red); the specific microglial marker ionized calcium binding adaptor molecule 1 (Iba-1) (red); and the activation indicator, vimentin (green). DAPI staining of nuclei shows total number of microglia. Arrowheads point to migratory morphologies. Scale bar, 20 µm; original magnification, ×63. Quantification of (A) tubulin-α, (B) F-actin, and (C) vimentin expression was performed via laser confocal microscopy and the ratio of overall fluorescence to cell numbers was calculated by digital image analysis using Image-Pro Plus version 5.1 software (Media Cybernetics, Inc.). Significant differences in mean fluorescence±SEM for n = 5 determinations/group was performed by one-way ANOVA and Tukey's post-hoc multiple comparisons where p<0.05 was considered significant.

Tubulin-α expression by microglia demonstrated 2 distinct morphologies; a resting cell morphology which was characterized by ramified, elongated, and rounded cells, and a migratory cell morphology consisting of polarized and tailing structures [54] (Fig. 4A). Migratory morphologies in virus-infected, single cell-type cultured microglia (MCG) predominated; however, elongated cells were substantially increased in microglia co-cultured with astrocytes (MCG-AST) without significant changes in tubulin-α expression among the treatment groups as determined by mean fluorescence intensity (Fig. 4B). Infection of microglia with HIV-1/VSV resulted in increased levels of F-actin polarized cells compared to uninfected microglia (Fig. 4A and C). Co-culture of infected microglia with astrocytes diminished levels of F-actin polarized microglia compared with infected microglia cultured in the absence of astrocytes (p<0.01). Iba-1 staining showed >98% of the identified cells were microglia (Fig. 4A). As demonstrated by increased mean fluorescence intensities, HIV-1/VSV infection of microglia increased expression of vimentin compared to uninfected microglia (n = 3, p<0.05) (Fig 4A and D). These levels were comparable to levels attained by activation of uninfected microglia with TNF-α. However co-culture of infected microglia with astrocytes diminished vimentin expression (n = 3, p<0.01, compared to infected microglia cultured alone) to levels attained by co-culture with uninfected microglia (n = 3, p>0.05).

### Astrocytes modulate microglial cell death

**Figure 5:**
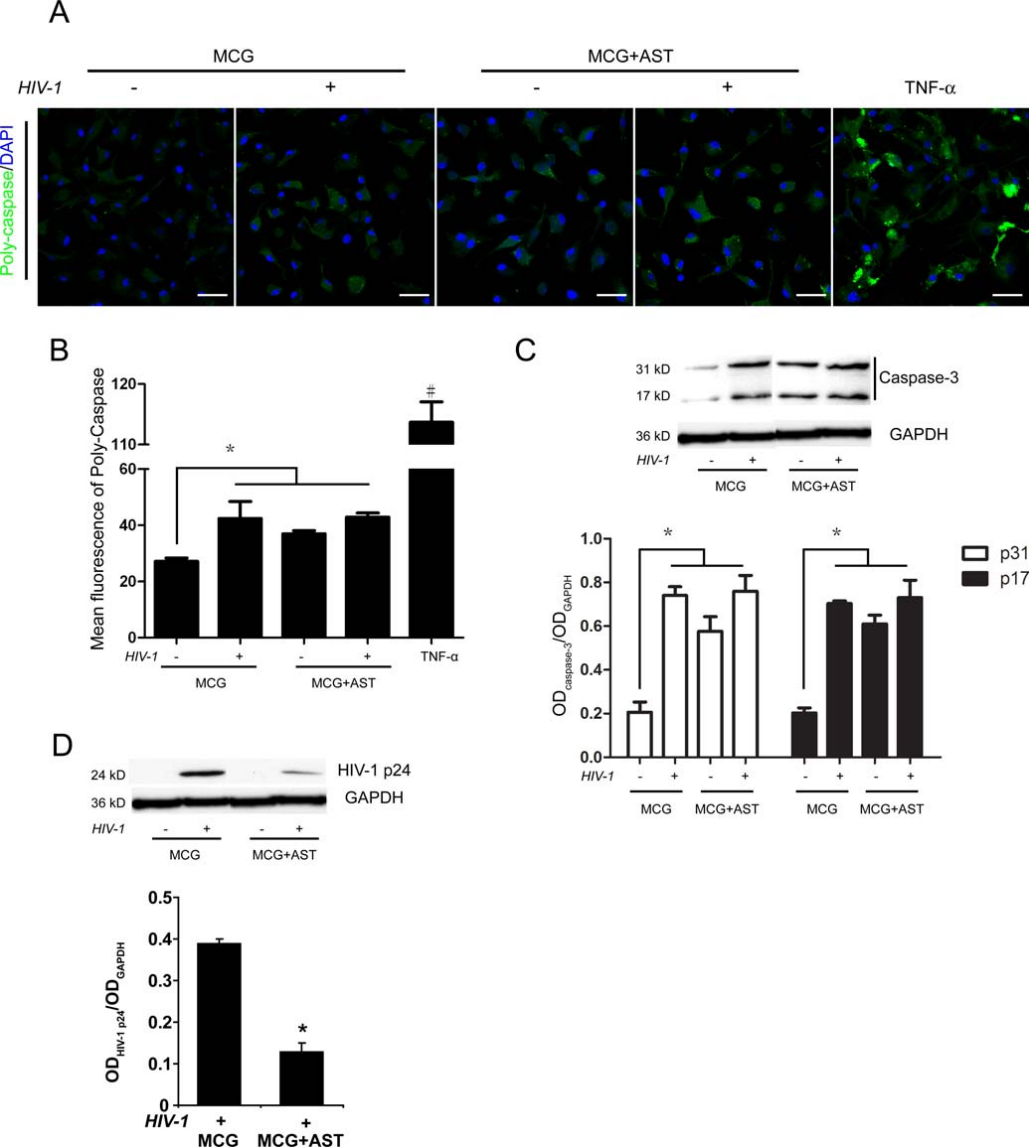
Caspase in HIV-1/VSV microglial infections. Uninfected or HIV-1/VSV infected microglia were cultured for 24 h in the absence (MCG) or presence of astrocytes (MCG+AST), Uninfected microglia cultured in the presence of 20 µg/ml TNF-α for 6 hours served as an activated microglia control. (A) Microglia were treated with a fluorescent-labeled inhibitor of caspases (FLICA; Immunochemistry Technologies, Bloomington, MN) to detect poly-caspases. Scale bar, 20 µm; original magnification, ×63. (B) Quantification of poly-caspase expression as detected with FLICA was achieved by fluorescence microscopy and digital image analyses using Image-Pro Plus version 5.1 software (Media Cybernetics, Inc.) to calculate the ratio of overall fluorescence due to FLICA staining to that obtained by DAPI stained nuclei. Differences in mean poly-caspase fluorescence±SEM for n = 5 determinations/group was assessed by one-way ANOVA and Tukey's post-hoc tests for multiple comparisons. *p<0.05, significant compared with uninfected single cultured microglia. ^#^p<0.05, significant compared with other 4 groups. (C) Western blot analysis of caspase-3 p 31 and p 17 levels showed trends consistent with FLICA analysis. Densitometric analysis of p 31 and p 17 levels were normalized to GAPDH levels and mean±SEM were determined for n = 5 determinations. Differences between means were determined by one way ANOVA and Tukey's post-hoc test for multiple comparisons where *p<0.05. (D) Western blot analysis showing HIV-1 p24 expression in cultures of infected microglia alone (MCG) or co-cultures with astrocytes (MCG+AST). Densitometric analysis of HIV-1 p24 levels were normalized to GAPDH levels and mean±SEM were determined for n = 3 determinations. Differences between means were determined by one way ANOVA and Tukey's post-hoc test for multiple comparisons where *p<0.01. HIV-1 p24 expression was reduced from infected microglia after co-culture with astrocytes (MCG+AST) compared to that of MCG.

We next sought to validate microglial cell death pathways, which were implicated by IPA network analysis, and used caspases as a protein marker for cell death induction. Multiple intracellular caspases were labeled with fluorescent-labeled inhibitor of caspases (FLICA) and mean fluorescence intensity (MFI) was quantified by confocal microscopy of uninfected or infected microglia cultured in the presence or absence of astroctyes. Microglia cultured with TNF-α was used as positive control for activated microglia. Minimal caspase expression was observed in uninfected microglia as determined by the low fluorescence signal, however treatment with TNF-α induced over a 3-fold increase in caspase levels (P<0.01) (Fig 5A and B). HIV-1/VSV infection of microglia and/or co-culture with astrocytes up-regulated caspase expression compared to uninfected microglia (n = 5, p<0.01). Notably, microglia in all astrocyte co-cultures showed higher caspase levels than the uninfected without cell-cell cultivation (Fig. 5A), however no significant differences in caspase expression as determined by FLICA stain and MFI could be discerned (Fig. 5B). On the other hand, caspase-3 expression as determined by Western blot was consistent with immunochemical assays showing increased levels of p 31 and p 17 in microglia after culture in the presence of HIV-1 and/or astrocytes and HIV-1 (Fig. 5C). Most importantly, co-culture with astrocytes significantly reduced levels of HIV-1 p24 expression by infected microglia compared to those cultured in the absence of astrocytes (n = 3, p<0.01) (Fig. 5D), suggesting that astrocytes suppress viral replication within infected microglia. Together, these results demonstrate the ability of astrocyte co-culture to activate cell death pathways in infected microglia, thus diminishing the capacity for productive viral replication.

### Astrocytes influence macrophage migratory responses

**Figure 6:**
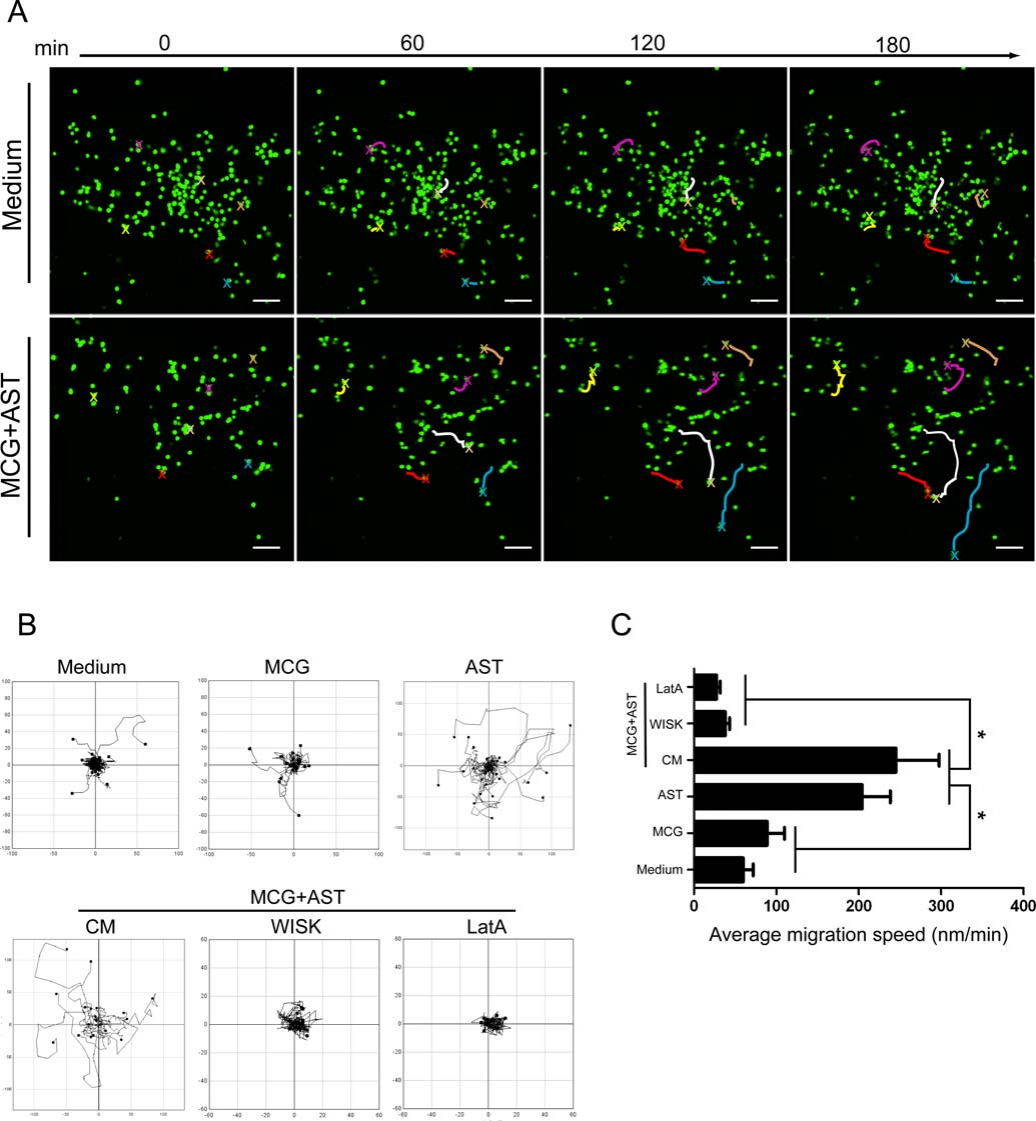
BMM mobility after exposure to micoglia/astrocvte conditioned media. Migration of CMFDA-labeled BMM was assessed for 3 h using confocal microscopy live imaging in μ-slide chemotaxis assays comparing fresh media with conditioned media (CM) from microglia alone (MCG), astrocytes alone (AST) or microglia-astrocyte co-cultures (MCG+AST). Inhibitors wiskostatin (WISK, 20 µM) and latrunculin A (LatA, 0.2 µg/ml) were utilized to inhibit BMM migration. (A) Representative migrating BMM cultured in fresh medium (Medium) or CM fro microglia-astrocyte co-cultures (MCG+AST) were tracked (X's in different color tracings) and evaluated at 60 min, 120 min, and 180 min. Scale bar, 10 µm; original magnification, ×10. (B) Plots of each individual cell migration were generated from tracking data acquired for 3 h cultures in medium (Fresh Medium), CM from microglia (MCG), astrocytes (AST), or astrocyte-microglia co-cultures (CM), and in the presence of WISK and LatA inhibitors. Data were acquired by laser confocal microscopy and ImageJ software (NIH) interfaced with the ManulTrack plugin. (C) Migration velocities for each individual cell track were determined with the Chemotaxis and Tool software tool (ibidi). Significant differences in the mean±SEM migration velocities for n = 50 determinations/group were assessed by two-tail Student's t test where *p<0.05 was considered significant.

Based on the capacity of HIV-1 infection to mobilize monocyte/macrophage migration to sites of neuroinflammation, we evaluated the capacity of microglia-astrocyte interactions to govern locomotion of bone marrow-derived macrophages (BMM). To assess the extent of migration by laser confocal microscopy live imaging, we monitored the mobility of 5-chloromethylfluorescein diacetate (CMFDA)-labeled BMM cultured in chemotaxis chambers with media alone or CM obtained from infected microglia in the presence or absence of astrocytes. Increased trafficking of BMM cultured in the presence of microglia-astrocyte CM was prevalent by 60 minutes after initiation of culture and migration speed peaked by 180 minutes post-initiation compared to medium control (Fig. 6A). Minimal BMM migration was observed in fresh media or microglial CM; however astrocyte and microglia-astrocyte CM induced robust levels of BMM migration (Fig. 6B). This level of BMM migration was attenuated by wiskostatin (WISK), a specific inhibitor of neural Wiskott-Aldrich syndrome protein (N-WASP)-mediated actin polymerization and by latrunculin A (LatA), an F-actin inhibitor. These results were consistent with the mean migration speed. BMM migrated at 59.7±12.1 nm/min (mean±SEM) in fresh media and 88.8±21.2 nm/min in microglial CM (Fig 6C). CM from astrocytes increased BMM migration speed to 204.4±34.0 nm/min and microglia-astrocyte CM increased migration to 245.3±52.4 nm/min (p<0.01 compared to speed with media or microglial CM). Treatment with WISK or LatA diminished the speed of microglia-astrocyte CM-induced BMM migration to 37.9±5.3 nm/min and 27.4±4.6 nm/min, respectively (p<0.01). These observations were independent of the release of macrophage chemotactic protein-1 (MCP-1), which showed distinct affects on macrophage mobility (our unpublished observations).

## Discussion

In the present study, we demonstrate that secreted products from astrocytes have a profound affect on the phenotype of HIV-1 infected microglia. This occurs, in measure, by the ability of astrocytes to modulate networks of MP death and cell migration. Specifically, astrocyte-microglial crosstalk leads to increases in microglial proteins affecting the assembly and projection of filaments and projections. Changes in severing and capping proteins lead to morphological changes in microglia consistent with an elongated resting phenotype. Coincident with these activities, astrocytes affect microglial metabolic activities and stimulate cell death pathways. Reduced HIV-1 p24 expression is coincident with a lowering of neurotoxic responses of infected microglia. Such complex cell-cell communication processes are likely linked to disease tempo and significance. These observations lead to the question as to how astrocytes control viral replication in brain MP and how astrocytes regulate ongoing neurotoxicity. To answer these questions, we used primary mouse cells as targets for HIV infection and a proteomic approach to explore astrocyte cross-talk with HIV infected microglia. Our results provide new insights into molecular mechanisms of astrocytes regulating HIV-infected microglia.

Proteomics is a powerful, high-throughput approach for study of cellular responses to biological stimuli at the protein level, but the utility of the method depends on the uniformity and reproducibility of the cellular systems under investigation. Although the present studies should ideally be conducted with human cells, primary astrocytes and microglia can only be obtained from abortive human fetal tissues and multiple tissue acquisitions from multiple donors are required. To ensure homogeneity of cellular targets in the present study, we elected to conduct this work with primary astrocytes and microglial cells obtained from a single inbred mouse strain. Rodent cells have been widely used in studies on the role of HIV Tat, gp120, and other viral proteins in neuroAIDS [55]–[59]. Since mouse or rat CD4 is not recognized as receptors for cellular entry by HIV, it has been generally assumed that mouse cells are resistant to HIV infection. Nonetheless, engagement of yet known viral receptors on rodent cells by HIV or soluble gp120 can affect cell signaling pathways and often cell death in neural cells [29], [60]. Recent studies indicate however that this restriction is operational primarily at the virus entry level. Primary mouse cells can be infected after circumventing the block to viral entry by using either HIV pseudotyped with the VSV-G protein [48], [49] or recombinant HIV expressing the MuLV gp80 envelope, EcoHIV [50]. EcoHIV was also shown to be infectious and neuropathogenic in normal immunocompetent mice [50]–[52], thus creating a model for studies of HIV pathogenesis in small animals. Cells from transgenic rats expressing human CD4 and CCR5 receptors, as well as the transgenic animals, can support infection by wild-type HIV [61]. Finally, we have recently employed mouse microglial cells productively infected with HIV through the use of VSV-G pseudotyped virus to explore immune control of HIV in the brains of immunocompetent mice [49]. The present work demonstrates further use of rodent cells as a suitable model system for study of aspects of HIV neuropathogenesis that require infection with intact HIV.

Astrocytes are well known to directly influence neuronal survival and modulate the microenvironment of the nervous system by affecting glutamate uptake and release, free radical scavenging, water transport, and production of cytokines, chemokines and nitric oxide [25], [62]–[66]. Our results showing that astrocytes attenuate neurotoxicity caused by HIV-1/VSV infected microglia secretions support their noted regulatory function to contain disease progression. This was previously confirmed by astrocyte-mediated neuroprotection during the early stages of CNS injury or microbial infection (reviewed in [67]). In support of this idea, we not demonstrate that exposure of astrocytes to HIV-infected microglia significantly reduces neuronal death. The astrocyte-induced neuroprotective effect was shown to be associated with alterations of the apoptosis signaling pathway by multiple mechanisms. First, astrocytes attenuated microglial death protein expression while affecting an up-regulation of cell growth and signal transduction proteins. This included the noted expression of glutathione S-transferase mu1 (GSTM1), enolase-α, preoxiredoxin 1, galectin-3 and PP2A. Surprisingly, most of these proteins were linked to reactive oxygen species production, such as NO by iNOS and hydrogen peroxide (H_2_O_2_). iNOS was previously shown to parallel the severity of HIV-associated neurocognitive disorders (HAND) [68], [69], and the addition of NO to HIV-infected cells enhanced viral replication [70], [71]. With respect to HIV-1 infection, Tat exposure of microglia leads to expression of iNOS as well as NO production [72]. Second, proteins such as preoxiredoxin 1 belongs to a family of enzymes that reduces hydroperoxides and play a major role in the clearance of low concentrations of H_2_O_2_ [73] and reduction of electrophilic neurocytotoxicity. GSTM1 has high detoxifying activity for 4-hydroxy-2-nonenal, the major hydroxylalkenal that is formed during peroxidation of polyunsaturated fatty acid and is highly cytotoxic to neuronal cells [74]. We demonstrated that astrocytes significantly down-regulate GSTM1 in HIV-infected microglia and attenuate HIV neurotoxicity by affecting NFκB signal pathways which reduce iNOS activity and NO radicals [75], [76]. Third, microglial NFκB pathway is attenuated by regulation of MARCKS, enolase-α, calmodulin, and protein kinase C and casein kinase substrate in neurons 2 (PACSIN2). MARCKS is a major protein kinase C (PKC) substrate in a variety of cells including brain MP [77]. Importantly, MARCKS interacts with the plasma membranes of macrophages [78], [79], neurons [80] and fibroblasts [81]. The unphosphorylated form of MARCKS binds to actin filaments, leading to cross linking and sequestration of membrane phospholipids, whereas phosphorylation by PKC abrogates membrane binding of MARCKS. MARCKS positively affects brain development and neuronal survival, cellular migration and adhesion, as well as endo-, exo- and phago-cytosis, and neurosecretion [78], [81], [82]. Our current study reveled that MARCKS was reduced in microglia infected with HIV-1. Enolase-α is a glycolytic enzyme described as a heat-shock protein in yeast and shown to be an early target of oxidative damage by carbonylation in different cell systems, ranging from yeast to humans [83], [84]. Interestingly, enolase-α significantly increases intracellular Ca^2+^, which leads to Bax translocation to the mitochondria and release of cytochrome c into the cytoplasm which correlates with the initiation of apoptosis and down-regulation in apoptotic nuclei [85]. Last, we found in our previous work that enolase-α was also reduced in HIV-infected microglia as well [86]. Heat shock proteins (HSPs), especially HSP-70, known as stress proteins, are induced upon cellular injury including hypoxia ischemia, heat trauma, neurodegenerative disorder, viral and bacterial infection, inflammation and oxidant injury [87] and have diverse functions including regulation of the redox state, modulation of protein turnover, and protection of CNS [88], [89]. HSPs can also protect cells from the consequences of protein misfolding and induce anti-apoptotic proteins, however, acute HIV infection results in increased HSP-70 mRNA and protein levels [90] and redistribution of HSP-70 on cell surface of the infected cells [91]. HSP-70 incorporates other HSPs, such as HSP-60, into the membrane of HIV-1 virions through Gag interactions [92], which can augment immune responses. A recent study demonstrated that HSP-70 also can protect astrocytes from cell death induced by HIV proteins [34]. Furthermore, astrocytes susceptible to neurotoxic processes and increased HSP-70 protein that may point to a possible pivotal role of HSP-70 in the signaling pathways of stress tolerance [93]. These discoveries are compatible with our current study. In this study, we also showed microglial biliverdin reductase, which converts biliverdin into antioxidant bilirubin [94] was up-regulated after infected microglia were co-cultured with astrocytes. Taken together, these results suggest astrocytes attempt to regulate microglial activation as well as productive HIV-1 infection via regulation of reactive oxygen species-induced cell death pathways. A possible mechanism for this regulation is the production of immunosuppressive factors, such as indoleamine 2,3-dioxygenase, as well as other redox enzymes, such as biliverdin reductase [95]–[98].

Mechanisms controlling over-activation represent a most important means to avoid inflammation-related CNS injury, which is a common factor associated with many severe neurodegenerative diseases, such as HAND, Parkinson's disease, and multiple sclerosis (MS) [99]–[101]. Thus, astrocytic regulation of microglial activation may be one of the reasons why HIV-1 p24 production was dramatically reduced when infected microglia were co-cultured with astrocytes. Astrocytes regulate gene expression, such as enhancing cell growth and signal transduction, to overcome HIV-infected microglia active. PP2A is a phosphoprotein reported to affect HIV-1 transcription and viral replication, and inhibition of PP2A enzymatic activity compromises Tat-induced HIV-1 transcription and viral production [102]. In addition, increase of intracellular PP2A activity enhances activation of HIV-1 promoter by phorbol myristate acetate (PMA), whereas inhibition of PP2A prevents its activation [103]. In this study, we observed both in DIGE and Western blot assays that microglia expressed significant less PP2A when co-cultured with astrocytes compared to its expression in the absence of astrocytes, regardless of microglial infection status. This implies that astrocyte-mediated restriction on HIV-1 replication in microglia is partly due to deregulation of microglial intracellular PP2A.

Most significant in this study was the capacity of astrocytes to alter the cytoskeletal protein network in viral infected-microglia. The current work extends previous observations made in our laboratories that actin and profilin rearrangement in human macrophages, along with exosomal secretion, affect viral replication and cytopathicity [104]. HIV-1 infection of microglia enhances F-actin and microtubule polarization which are both essential for cell mobility. These responses can be modulated by astrocytes via eliciting changes in the cytoskeleton protein network associated with F-actin protein transformation. In contrast, astrocytes affect HIV-infected microglial cytoskeleton proteins that could lead to assembly of filaments and formation of plasma membrane projections. For example, down-regulation of APC, MAPRE1 and TPM3 were observed which are reported to increase the microtubule polymerization [105], [106]. Infected microglia when co-cultured with astrocytes, up-regulated VIL2, WASF2, and PACSIN2, all having been shown to positively affect plasma membrane projections [107]–[110]. Recently, reports suggest that MARCKS protein mediates regulation of the actin cytoskeleton directly via binding and possibly cross-linking PIP2 dependent proteins [77]. Indeed, BMM migration was enhanced by astrocytes. This was linked to actin polymerization, but may also be linked to proteins that cap growing filaments, sever existing filaments from older proteins, and control the availability of activated actin monomers [111]. The latter is most important for cells to form necessary polarities required for locomotion; the lack of these proteins can lead to elongation of actin filaments and a resting status instead of polarization and migration. This was shown by comparison studies of proteomic and immunochemical assays. The former studies showed that microglia in co-culture with astrocytes exhibited a network merely increasing the polymerization of actins without differential expression of severing proteins supporting significant increases seen in elongated microglial morphologies. This loss of the typical ramified morphology for resting microglia after co-culture with astrocytes and inhibition of PP2A activity was reported by others [112]. These findings support our results demonstrating a dramatic down-regulation of microglial PP2A associated with profound changes in cell morphology.

In conclusion, together with changes in cytoskeletal and cell death pathways, modulation of glutamate transport and anti-oxidant proteins were previously found in virus-infected astrocytes or in microglia exposed to astrocyte secretions [113]–[115]. For example, the astrocyte elevated gene (AEG)-1 is inducible in infected astrocytes by HIV-1 and TNF-α. AEG-1 down-regulates the expression of the glutamate transporter EAAT and as such, is directly linked to glutamate-induced excitotoxic damage to neurons during progressive HIV infection [116], [117]. Moreover, astrocytes also regulate oxidative damage, a critical component of neuroinflammatory activities, seen throughout the breadth of neurodegenerative diseases [118]–[120]. Functions of oxidative damage for which astrocyte-mediated regulation is attributable include abnormal protein clearance, depletion of the cellular redox-balance, and interference with the cell cycle. These, taken together ultimate affect neuronal survival. Therefore, identification of specific proteins protected from oxidation is an important means to best understand the types of communication pathways developed in the current report. Previous works demonstrated that β-actin, calreticulin precursor protein, and synovial sarcoma, X breakpoint 5 (SSX5) isoform A were increased in oxidative modifications by astrocytes exposed to Tat [35]. All together our results provide new insights into astrocyte-mediated protection against neurotoxicity seen during HIV-1 microglial infections.

## Materials and Methods

### Primary mouse microglia, astrocyte and neuron isolation

Embryonic day 18 old fetuses were harvested from terminally anesthetized pregnant C57BL/6 mice. Animals were maintained in sterile microisolator cages under pathogen-free conditions and bred in accordance with ethical guidelines for care of laboratory animals at the University of Nebraska Medical Center as set forth by the National Institutes of Health. Cerebral cortices were isolated and digested using 0.25% trypsin (Invitrogen, Carlsbad, CA) in PBS for 15 mins at 37°C. Single-cell suspensions were cultured under various conditions to differentiate into neurons, microglia (MCG) and astrocytes (AST). For neurons cortical isolates were seeded at a density of 1.2×10^5^ cells/well on poly-D-lysine coated cover slips, placed in 24-well plates and maintained for 10 days in neurobasal medium supplemented with 2% B27 (Invitrogen), penicillin/streptomycin and 0.5 mM L-glutamine (Invitrogen). Primary neurons were determined to be >98% as determined by staining for expression of microtubule-associated protein-2 (MAP-2) and with DAPI for nuclei, while <2% of the cells expressed glial fibillary acidic protein (GFAP) (data not shown). For microglia, cortical isolates were cultured for 14 days in DMEM supplemented with 10% FBS, 2 mM L-glutamine, 1% penicillin/streptomycin (all from Invitrogen), and 2 µg/ml macrophage colony stimulating factor (MCSF) (a generous gift of Wyeth Inc., Cambridge, MA, USA). For astrocytes, cortical isolates were cultured for 14 days in DMEM supplemented with F12, 10% FBS, 2 mM L-glutamine, and 1% penicillin/streptomycin (all from Invitrogen).

### Single culture and transwell co-culture

For single cell-type cultures in 6-well plates, microglia were seeded at 2×10^6^ cells/well and astrocytes at 1×10^6^ cells/well. For co-culture, uninfected or infected microglia were seeded in 6-well culture plates at 2×10^6^ cells/well and cultured for 3 d before adding transwell inserts containing uninfected astocytes at 1×10^5^ cells/insert. Importantly, conditioned media (CM) prepared from either single cell-type or co-cultures were mixed and utilized at a 1∶1 ratio of microglial CM and astrocyte CM from the above cultures.

### Viruses and infection

Vesicular stomatitis virus (VSV) pseudotyped HIV-1 strain YU2 was used to circumvent receptor HIV-1 co-receptor requirements and infect mouse cells. Cells were infected with HIV-1/VSV at a final concentration of 1 pg HIV-1 p24 per cell for 24 h prior to rinsing and were cultured for 7 days before use. Greater than 98% of microglia were infected as determined by immunohistochemical staining for HIV-1 p24 (data not shown).

### TUNEL assay

Terminal deoxynucleotidyl transferase-mediated biotinylated UTP nick end labeling (TUNEL) was performed using the in situ cell death detection kit, AP (Roche Applied Science, Indianapolis, IN) according to the manufacturer's instructions. Briefly, neurons were fixed with 4% paraformaldehyde in PBS (pH 7.4) and permeabilized with 0.1% TritonX-100 in 0.1% sodium citrate. Cells were subsequently labeled with TUNEL working solution. Apoptotic cells were identified as green fluorescent TUNEL positive cells by fluorescence microscopy, and were normalized to total number of cells as determined by DAPI nuclear staining.

### Protein sample preparation

Cells were washed in PBS, harvested and lysed in 200 µl cell lysis buffer containing 7 M urea, 2 M thiourea, 4% 3-[(3-cholamidopropyl)dimethylammonio]-1-propanesulfonate (CHAPS) (pH 8.5) and 1× protease inhibitor cocktail (Biovision, Mountain View, CA). Cell lysates were sonicated at 25 W for 3 pulses at 3 sec per pulse (W-225 Sonicator, Heat Systems-Ultrasonics, Inc., Farmingdale, NY). To remove impurities and sample concentration, cell lysates were treated with a 2D Clean Up Kit (GE Healthcare, Piscataway, NJ) according to the manufacturer's instructions. Protein concentration was determined with the 2D Quant Kit (GE Healthcare).

### Difference gel electrophoresis (DiGE) and image analysis

Fifty micrograms of protein sample from uninfected or HIV-1/VSV-infected microglia were respectively labeled with 400 pmol of Cy3 or Cy5 (CyDye Minimum Labeling kit, GE Healthcare). Twenty-five micrograms of unlabeled protein from each sample were mixed and labeled Cy2 (GE Healthcare) to serve as internal standards. The resulting Cy2-, Cy3-, and Cy5-labeled cells were pooled and mixed with rehydration buffer (7 M urea, 2 M thiourea, 2% CHAPS, 50 mM DTT, 1% Pharmalyte (pH 3–10NL). Labeled protein samples were loaded onto gel strips with an immobilized pH gradient (24 cm; pH 3–10 NL) and the first-dimension separation was achieved by isoelectric focusing (IPGphor II, GE Healthcare) for 1 h at 500 V, 1 h at 1000 V, and 3 h at 8000 V. The focused strips were treated with equilibration solution [6 M urea, 30% glycerol, 2% sodium dodecyl sulfate (SDS), 50 mM Tris (pH 8.8)] containing 100 mM DTT for 10 min. Thereafter, strips were alkylated with 100 mM iodacetamide (Sigma-Aldrich, St Louis, MO) in equilibration solution for 10 min. Immediately after, gel strips were placed on top of a 10–20% polyacrylamide gradient reducing gel and 2^nd^ dimension separation was achieved by electrophoresis at constant 5 mA for the initial 30 min and 12 mA for 14 h. For visualization of protein spots, gels were read with a 2D Master Imager (GE Healthcare) at excitation wavelengths of 488 nm, 520 nm, and 620 nm and emission wavelengths of 520 nm, 590 nm, and 680 nm to detect Cy2-, Cy3-, and Cy5-labeled proteins, respectively. The relative protein amount for each spot was determined by digital quantification using DeCyder-DIA software (GE Healthcare). Protein levels exhibiting greater than 1.5-fold changes above or below relative amounts after normalization were considered candidates for further analyses as an arbitrary cut off.

### Spot picking and in-gel tryptic digestion

Protein spots within 2 mm^2^ fragments were robotically picked from the gel using the Ettan™ Spot Picker (GE Healthcare) and destained for 1 h at room temperature using 100 µl of 50% acetonitrile (ACN)/50 mM NH_4_HCO_3_. Gel fragments were dried and incubated with 0.25% trypsin/10 mM NH_4_HCO_3_ (Promega) overnight at 37°C. Peptides were extracted by washing gel pieces twice with 0.1% trifluroacetic acid (TFA) and 60% ACN.

### Mass spectrometry

All samples were purified using ZipTip® (Millipore) prior to mass spectrometric analysis. Peptide samples were resuspended in 0.1% formic acid/HPLC-grade water and analyzed by LC/MS/MS (LCQ DECA XPPlus, ThermoElectron, Inc. Waltham, MA) and MALDI-Tof-Tof mass spectrometry (ABI 4800, Applied Biosystems, Foster City, CA). Proteins were identified from the NCBI database interfaced with BioWorks 3.1SR software (ThermoElectron). Protein identifications scoring greater than 3000 by the Unified Score scale and greater than 50% on ion score were considered for further analyses. For MALDI-Tof-Tof, peptide samples in α-cyano-4-hydroxycinnamic acid (CHCA; Sigma-Aldrich) matrix were spotted on to Opti-TOF® 384 well MALDI plate inserts (Applied Biosystems). The mass profile analyzed by the MALDI-TOF-TOF mass spectrometer was searched in Mascot database assisted by GPS Explorer software (Applied Biosystems) and only significant protein identifications (p<0.05) were considered for further analyses.

### Ingenuity pathway analysis

A data set containing protein identifiers and corresponding expression values was uploaded into in the application Ingenuity Pathways Knowledge Base. Each protein identifier was mapped to its corresponding gene in the database and networks of these focus genes were algorithmically generated based on their connectivity. The Functional Analysis identified the biological functions and/or diseases that were algorithmically significant to the data set. The graphical representation of the molecular relationships between genes/gene products are represented as nodes, and the biological relationship between two nodes is represented as an edge (line). All edges are supported by at least 1 reference from the literature, from a textbook, or from canonical information stored in the Ingenuity Pathways Knowledge (Ingenuity® Systems, www.ingenuity.com).

### Immunohistochemistry

Cells adhered to cover slips were fixed and permeabilized with acetone and methanol (ratio 1∶1, at −20°C) for 10 min, and nonspecific activity was blocked by incubation of fixed cells in 6% BSA/PBS. Blocked samples were reacted with 250 µl of diluted corresponding primary antibody which included mouse anti-neuron-specific nuclear protein (NeuN) (1/100; Chemicon, Temecula, CA), rabbit anti- microtubule associate protein-2 (MAP-2) (1/1000; Chemicon), rabbit anti-tubulin-α (1∶1000; Novus, Littleton, CO), rabbit anti-ionized calcium binding adaptor molecule 1 (Iba-1) (1∶500, Wako, Richmond, VA), and mouse-anti-vimentin (1∶1000; Dako, Carpenteria, CA). F-actin was detected with rhodamine-conjugated phalloidin (Invitrogen). Primary antibody reacted cells were incubated with 250 µl of the appropriate diluted goat anti-mouse or goat anti-rabbit Abs secondary antibodies (1∶250; Invitrogen) conjugated to Alexa-488 or Alexa-568. Cells were mounted using anti-fade Pro-Long Gold mounting reagent (Invitrogen) and examined with a Zeiss LSM 510 META NLO microscope (Zeiss MicroImaging, Inc., Thornwood, NY).

### Western Blotting

For Western blots, 10 µg of protein from each total cell lysate was electrophorectically separated on SDS-PAGE and electrotransfered to polyvinylidene membranes (Roche). Membranes were incubated overnight at 4°C with specific primary antibodies including, mouse anti-PP2A (1∶500; Cell signaling, Danvers, MA), mouse anti-vimentin (1∶500; Dako), rabbit anti-tubulin-α (1∶1000; Novus), rabbit anti-biliverdin reductase (1∶5000; Abcam, Cambridge, MA), rabbit anti-enolase-α (1∶1000; Santa Cruz, Santa Cruz, CA), mouse anti-calmodulin (1∶1000; Abcam), mouse anti-HIV-1 p24 (1∶500; Dako) or rabbit anti-caspase-3 (1∶1000; Cell signaling). Detection of reacted primary antibodies was achieved with horseradish peroxidase (HRP)-conjugated goat anti-mouse (1∶10,000) or goat anti–rabbit (1∶10,000) IgG antibodies (Jackson Immunoresearch, West Grove, PA.) HRP activity was visualized by an enhanced chemiluminescence detection procedure (Pierce, Rockford, IL).

### Chemotaxis assay

BMM were isolated from femur bone marrow of C57BL/6 mice, and were dissociated into single cell suspensions. Bone marrow cells were seeded to Teflon-coated flasks at 2×10^6^ cells/ml in DMEM supplemented with 10% FBS, 2 mM L-glutamine, 1% penicillin/streptomycin (complete DMEM), and 2 µg/ml of MCSF (a gift from Wyeth) and cultured for 7 days. For chemotaxis assay, BMM were labeled with 10 µM CMFDA (Invitrogen) in DMEM at 37°C for 40 min, washed, and adjusted to 2.5×10^6^ cells/ml in complete DMEM. CMFDA-labeled cell suspensions were introduced into the μ-slides (ibidi, LLC, Verona, WI) in 10 µl per chamber and cultured for 35 min in the humid cell incubator at 37°C to allow cell attachment. Fresh or conditioned media (CM) were loaded to the two reservoirs on both sides of each chamber and cells were imaged for 3 h using a Nikon Swept-Field laser confocal microscope (Nikon Instruments, New York, NY). Images were analyzed using different plugins interfaced with ImageJ 1.38× software (Rasband, W.S., ImageJ, U. S. National Institutes of Health, Bethesda, Maryland, USA, http://rsb.info.nih.gov/ij/, 1997–2007). Migration co-ordination data for each observed cell was acquired with the ManulTrack plugin (Fabrice Cordelières, Institut Curie, Orsay, France). Chemotaxis plots and migration velocities of each cell were determined with the Chemotaxis and Migration Tool (ibidi).

### Statististics

All resulting data were analyzed for statistical significance either by two-tail Student's t-test or one-way ANOVA with Tukey's post-hoc multiple comparisons using GraphPad Prism version 4.0 (GraphPad Software, Inc., San Diego, CA). P<0.05 was deemed significant.
